# Current concepts for the diagnosis and management of eosinophilic granuloma of bone

**DOI:** 10.1007/s10195-016-0434-7

**Published:** 2016-10-21

**Authors:** Andrea Angelini, Andreas F. Mavrogenis, Eugenio Rimondi, Giuseppe Rossi, Pietro Ruggieri

**Affiliations:** 1 Department of Orthopedics, University of Bologna, Istituto Ortopedico Rizzoli, Via Pupilli, 40136 Bologna, Italy; 2 First Department of Orthopaedics, Athens University Medical School, ATTIKON University Hospital, Athens, Greece; 3 Department of Radiology and Interventional Angiographic Radiology, Istituto Ortopedico Rizzoli, Bologna, Italy; 4 Department of Orthopedics and Orthopedic Oncology, University of Padova, Padova, Italy

**Keywords:** Eosinophilic granuloma, Methylprednisolone injection, Spine, Extremities, Bone tumors

## Abstract

This review summarizes current concepts in the diagnosis and management of the patients with eosinophilic granuloma. Given the benign biology, the clinical course, and the pediatric group of patients that this condition more commonly affects, a treatment approach that carries a lower risk of complications while ensuring a successful cure is desirable. Variable treatment options have been reported with satisfactory results and a recurrence rate of less than 20 %. In this setting, symptomatic lesions that are accessible in the spine or the extremities may be treated with intralesional methylprednisolone injection after tissue biopsy for histological diagnosis.

## Introduction

Langerhans-cell histiocytosis is a rare disease involving clonal proliferation of Langerhans cells [1, 2]. It is part of a group of clinical syndromes called histiocytoses, which are characterized by an abnormal proliferation of histiocytes (an archaic term for activated dendritic cells and macrophages). These diseases are related to other forms of abnormal proliferation of white blood cells, such as leukemias and lymphomas. The disease, previously known as histiocytosis X, was renamed in 1985 by the Histiocyte Society as Langerhans-cell histiocytosis because of the proliferation of Langerhans-cells. The spectrum includes localized-to-bone eosinophilic granuloma, and the rare multisystem syndromes Hand–Schüller–Christian disease and Abt–Letterer–Siwe disease; the manifestations range from isolated bone lesions to multisystem disease [1, 2].

## Eosinophilic granuloma of bone

Eosinophilic granuloma is a rare, benign tumor-like disorder characterized by clonal proliferation of antigen-presenting mononuclear cells of dendritic origin known as Langerhans cells [1, 2]. It is the most common manifestation of Langerhans-cell histiocytosis (60–80 % cases), accounting for less than 1 % of all bone tumors [3]. In 80 % of cases it affects children and adolescents [4, 5]. It can affect any bone in the skeleton; however, bone lesions are more common in the skull, mandible, spine, ribs, and long bones; the femur, humerus and clavicle are the most frequent sites [6]. The pathogenesis is unclear; viruses such as Epstein-Barr and human herpes virus-6, bacteria, and genetic factors have been implicated [3, 7, 8]. An immunological dysfunction including an increase of certain cytokines such as interleukin-1 and interleukin-10 in affected patients has also been reported; familial occurrence is very rare [1, 9]. In the spine, eosinophilic granuloma accounts for 6.5–25 % of all spinal bone tumors [5, 10–16]. The most common location is the thoracic spine followed by the lumbar and the cervical spine [10, 15, 17–20]. Clinical symptoms are often severe and depend on spinal location [14, 15, 20]. The most common include back or neck pain, tenderness to spinal palpation and restricted range of motion, or torticollis; spinal instability and neurological symptoms are uncommon [5, 15, 21–25]. In the extremities, most lesions are diaphyseal [7]. The physical examination of the child may be essentially normal. Laboratory findings are usually non-specific except for a moderate and inconsistent rise in erythrocyte sedimentation rate.

## Imaging

The typical radiographic appearance of eosinophilic granuloma of the extremities is a punched-out lytic-bone lesion without reactive sclerosis. In most cases, a hypervascularized soft-tissue mass surrounds the affected bone [26, 27]. The radiographic differential diagnosis should include plasmacytoma, multiple myeloma, osteochondritis, tuberculosis or osteomyelitis. In the spine, imaging studies may reveal variable vertebral involvement, ranging from isolated lytic lesions to a more significant vertebral collapse that involves the pedicles and posterior vertebral elements (vertebra plana), peridural spread and paraspinal soft tissue components [20, 25]. Although eosinophilic granuloma is the most common cause of vertebra plana, this finding can also be found in Ewing’s sarcoma, lymphoma and other sarcomas, infections such as tuberculosis, and osteogenesis imperfect [28, 29]. In favor of the eosinophilic granuloma are the isolated spinal disease, the lack of constitutional symptoms, and minimal laboratory abnormalities [28]. Cervical spine eosinophilic granuloma more often manifests with osteolytic lesions, rather than vertebra plana [18, 20, 25, 30].

## Diagnosis

Tissue biopsy for histological diagnosis is necessary when clinical and radiological manifestations are ambiguous, and the lesions are symptomatic [5]. CT-guided biopsy for eosinophilic granuloma has been effective for histological diagnosis, with low morbidity and a diagnostic accuracy of 70–100 % [5, 31–38]. Although anecdotally excellent results with biopsy alone have been previously reported for patients with eosinophilic granulomas [39], biopsy should not be considered as a strategy for treatment of these patients but rather as a step to confirm diagnosis [26, 32, 33, 36–38, 40–42].

## Management

Various treatment options have been reported for eosinophilic granuloma of bone, including observation and immobilization, indomethacin administration, methylprednisolone injections, radiofrequency ablation, local excision and curettage with or without bone grafting, chemotherapy and irradiation; results have been reported as satisfactory with a recurrence rate of less than 20 % [11, 13, 14, 32–34, 43–48]. In general, the treatment of typical solitary lesions in asymptomatic patients is conservative [16, 20, 25]. In patients with mild neurological deficits from solitary eosinophilic granulomas of the spine, immobilization and radiation therapy has been reported [48]. Low-dose radiation therapy is advocated by some authors to be effective in the healing of lytic lesions and limiting disease progression [24]; others argue that radiation therapy may damage endochondral growth plates and limit bone healing and reconstitution [49, 50], or lead to secondary radiation-induced morbidity such as post-radiation sarcomas and myelitis [5, 51]. Although no clear correlation between the degree of vertebral collapse and the degree of neurological symptoms has been observed [25], in patients with severe pain and restriction of range of motion, and/or persistent spinal subluxation and neurological symptoms, surgical treatment is required [12, 13, 15, 19, 20]. Chemotherapy is not recommended for solitary eosinophilic granuloma, and should be reserved for systemic involvement [13, 20, 48], or as initial therapy in children with solitary lesions in locations that preclude safe and complete surgical resection [52].

Since eosinophilic granuloma in children is known to resolve spontaneously with time, observation alone or biopsy alone to confirm the diagnosis have also been recommended as a treatment strategy [39]. A previous study reported spontaneous resolution without recurrence of the lesions in six skeletally immature patients that had biopsy followed by observation alone (open biopsy in three and percutaneous in three), suggesting the intriguing possibility that surgery may result in a higher rate of recurrence than less aggressive procedures [39]. We concur that biopsy may have an effect on bone healing and eosinophilic granuloma lesions reconstitution [39]. However, we disagree that patients, especially children with symptomatic bone lesions should be left alone to let the disease take its natural course without a histological diagnosis. Moreover, although solitary eosinophilic granuloma is considered a benign lesion, without treatment, the time required for resolution is unpredictable and can be associated with significant morbidity secondary to unremitting pain, restricted activity, growth disturbance, or pathological fracture [26, 47]. Therefore, we recommend that these patients should undergo biopsy for histological diagnosis, and treatment is then considered [36–38].

## Methylprednisolone injection

**Fig. 1:**
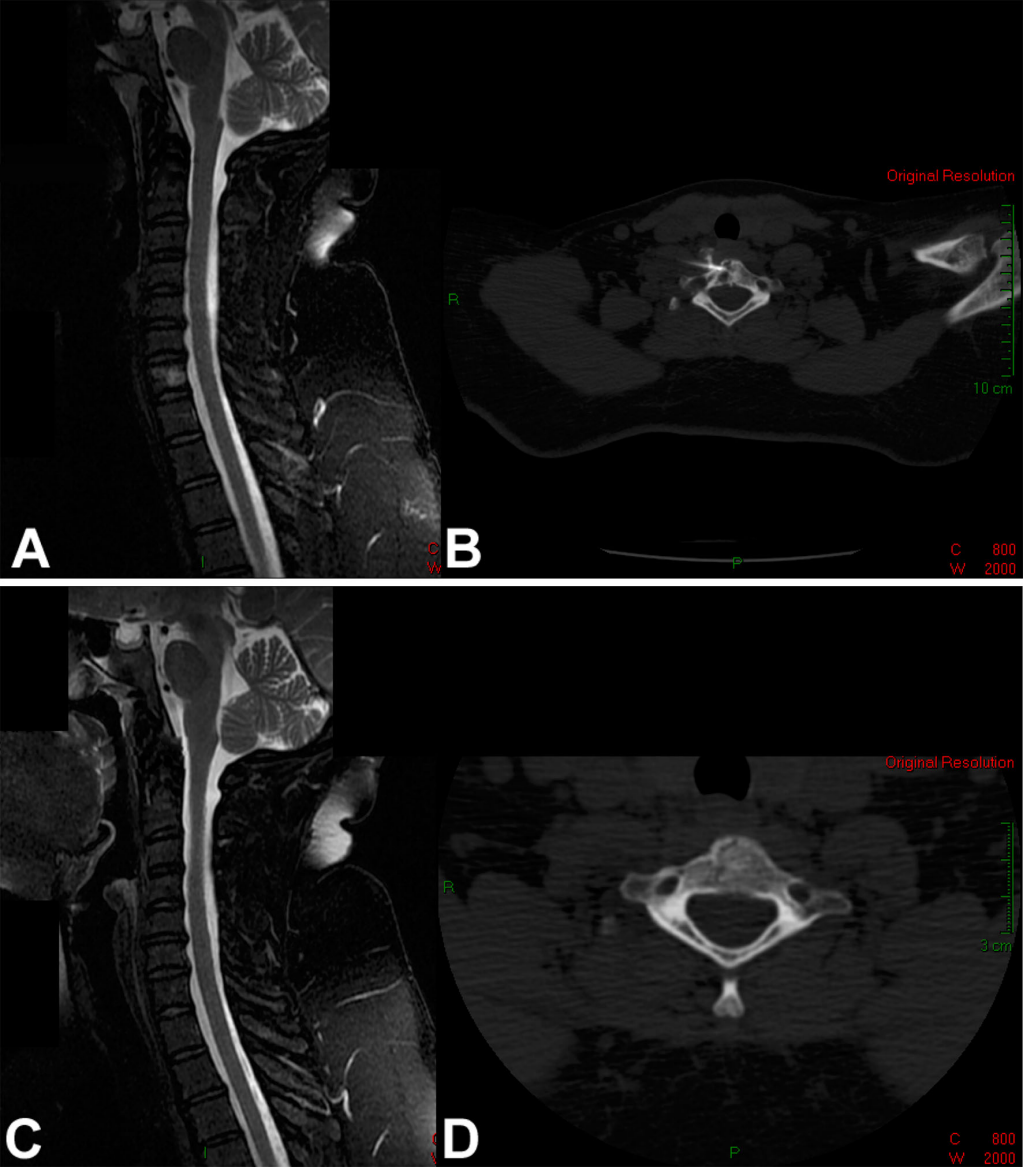
**a** Sagittal T2-weighted MRI with fat suppression of the cervical spine of a 43-year-old woman with a painful osteolytic lesion of the C7 vertebral body. **b** CT-guided frozen section biopsy showed eosinophilic granuloma; intralesional methylprednisolone injection was performed. **c** Sagittal T2-weighted MRI with fat suppression. **d** Axial CT scan show complete reconstitution of the lesion 4 years after diagnosis and treatment

**Fig. 2:**
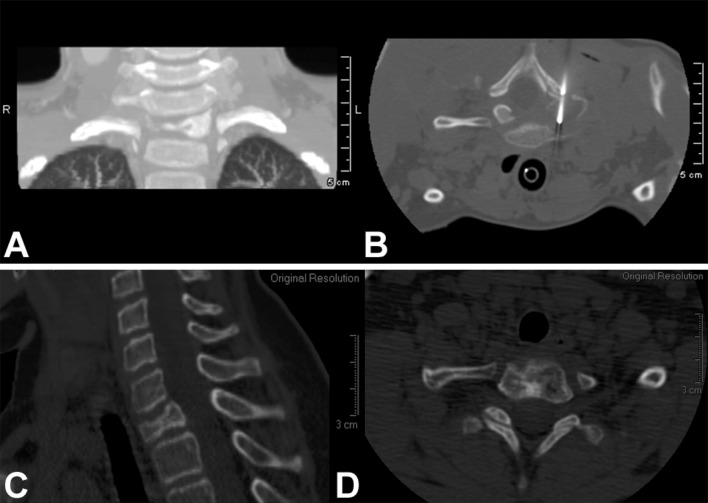
**a** Coronal CT scan of the cervical spine of a 5-year-old boy with a painful osteolytic lesion of the C7 vertebral body. **b** CT-guided frozen section biopsy showed eosinophilic granuloma; intralesional methylprednisolone injection was performed. **c**, **d** Sagittal (**c**)and axial (**d**) CT scans showing complete reconstitution of the lesion 5 years after diagnosis and treatment

**Fig. 3:**
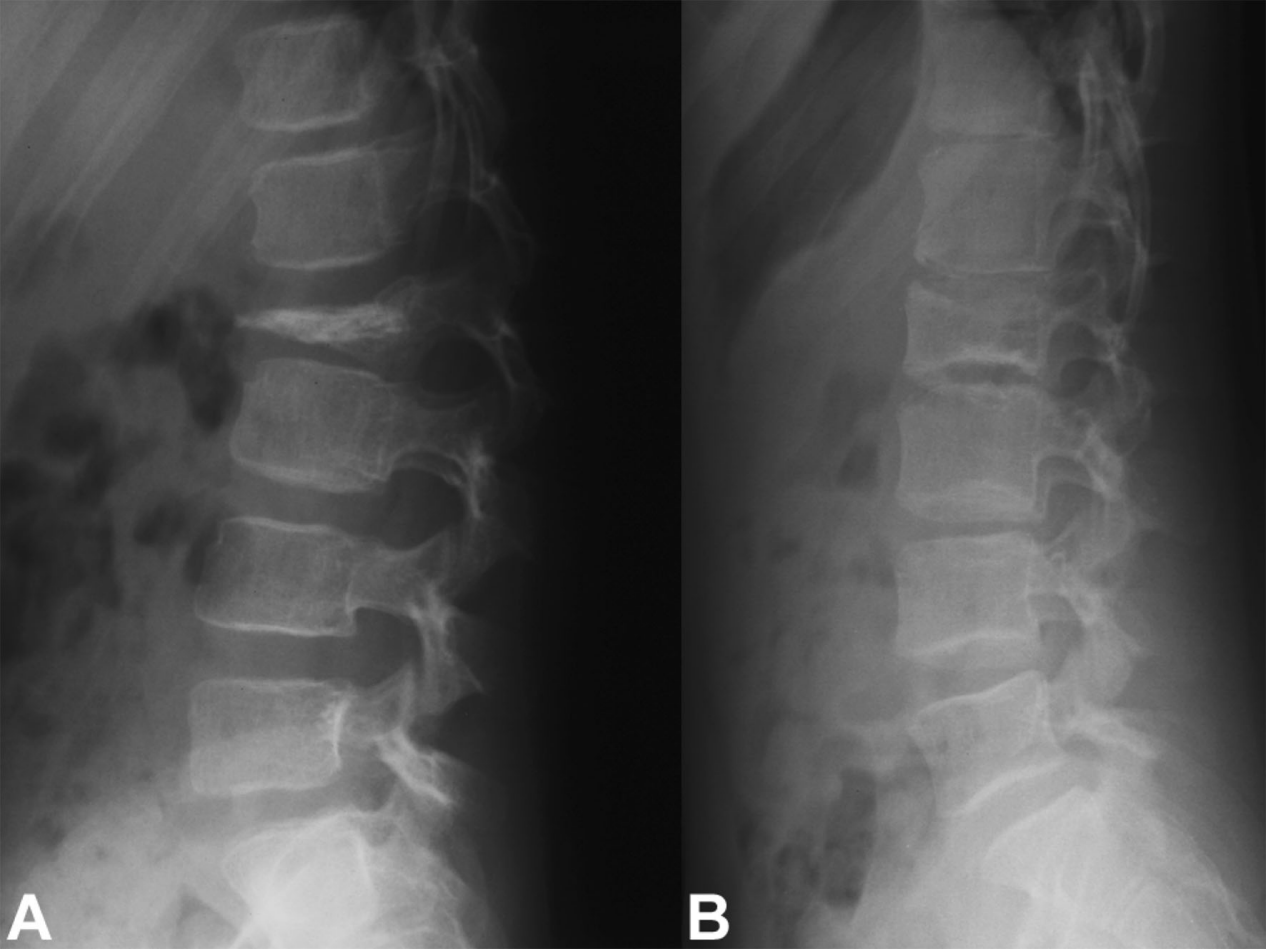
**a** Lateral radiograph of the lumbar spine of a 6-year-old girl with a painful osteolytic lesion of the L2 vertebral body with vertebral plana deformity. CT-guided frozen section biopsy showed eosinophilic granuloma; intralesional methylprednisolone injection was performed. **b** Lateral radiograph of the lumbar spine shows complete reconstitution of the lesion 7 years after diagnosis and treatment

**Fig. 4:**
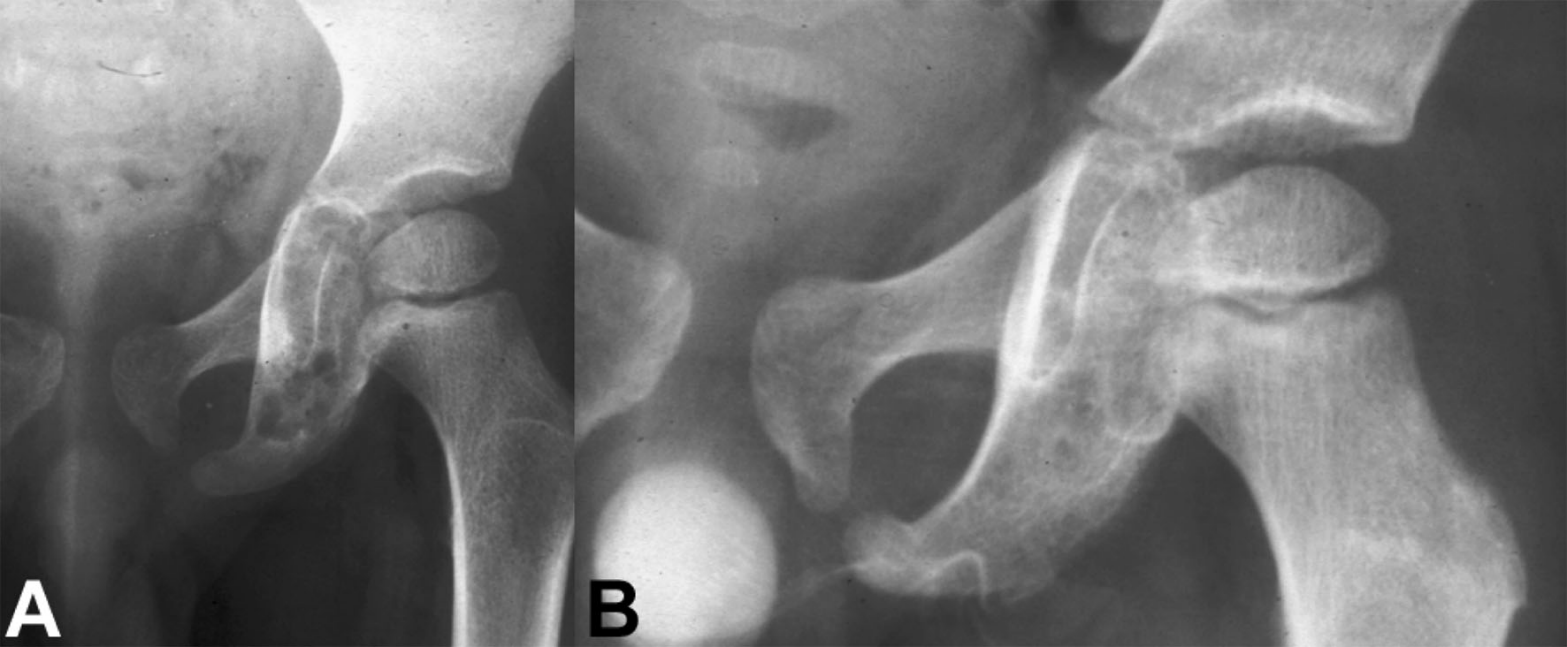
**a** Anteroposterior radiograph of the pelvis of a 6-year-old boy with a painful osteolytic lesion at the left ischial ramus. CT-guided frozen section biopsy showed eosinophilic granuloma; intralesional methylprednisolone injection was performed. **b** Anteroposterior radiograph of the pelvis shows complete reconstitution of the lesion 1 year after diagnosis and treatment

Given the benign biology and clinical course of eosinophilic granuloma and the pediatric group of patients that this condition more commonly affects, a treatment approach that carries a lower risk of complications while ensuring a successful cure is desirable. In this setting, symptomatic lesions that are accessible in the spine (Figs. 1, 2, 3) or the extremities (Fig. 4) may be treated with intralesional methylprednisolone injection after tissue biopsy for histological diagnosis [8, 26, 31–33, 36–38].

Langerhans histiocytosis, as a systemic disease, appears to be one of the most tissue-destructive syndromes, able to induce multiple and grossly apparent lytic lesions involving many organs and the bones. Since this lytic activity cannot be connected with a neoplastic nature of the disease, one can reasonably assume that histiocytosis X cells induce bone resorption in eosinophilic granuloma through their ability to secrete locally tissue-lytic factors such as interleukins and prostaglandins [53]. Several in vitro studies have demonstrated the production of interleukins (IL) such as IL-1 and prostaglandins (PG) such as PGE2 and PGD2 by suspensions of Langerhans cells [53, 54]. Although definitive proof that corticosteroid injection is responsible for the observed response is difficult to obtain, the inhibition of IL-1-induced bone resorption and prostaglandin production by methylprednisolone [55] may account for its clinical and radiographic effect. Previous studies on the treatment of certain osteolytic lesions including bone cysts, aneurysmal cysts, eosinophilic granulomas and nonossifying fibromas showed that the results obtained through the introduction of methylprednisolone acetate in crystals were better than those obtained by using other corticosteroids with topical action [40]. This is because it is a microcrystalline suspension of acetate of methylprednisolone that is relatively insoluble and, therefore, has a prolonged pharmalogical effect [40]. A particular dosage for eosinophilic granulomas cannot be recommended. The amount of methylprednisolone acetate injected was established empirically on the basis of the size of the lesion. A minimum of 40 mg for small lesions involving less than half the diameter of the involved bone, and up to 160 mg for large lesions of the pelvis has been recommended [36–38].

Scaglietti et al. [40] first reported the use of intralesional methylprednisolone injection for eosinophilic granuloma, with excellent results, and recommended the injection of methyl-prednisolone as the treatment of choice. Subsequently, similar clinical and radiographic results have been described in case reports and small series of patients with solitary and polyostotic lesions involving craniofacial and long bones [27, 33, 56–58]. The benefit of intralesional methylprednisolone injection compared with other methods is that it promotes early relief of pain and predictable osseous healing [31, 34]. The results of treatment, either as an adjunct or as primary treatment have been comparable to other treatments [26, 31–33, 40]. Others reported that intralesional methylprednisolone injection adds little in children [33, 39]. However, in patients with symptomatic lesions, treatment is required. In view of the usually benign clinical course of the disease, in these patients a simple, minimally invasive, outpatient treatment with a low rate of complications such as CT-guided intralesional methylprednisolone injection may be considered the treatment of choice [36–38].

Complications such as femoral osteomyelitis [32] and obstructive hydrocephalus have been reported after methylprednisolone injections for eosinophilic granulomas [33]. However, in general, the morbidity associated with the procedure has been negligible, even when relatively inaccessible regions of the spine or pelvis are involved [31]. The ability of the involved bone to reconstitute after methylprednisolone injection is believed to be due to the fact that the disease affects children before skeletal maturity so that the pubertal growth spurt provides sufficient time for adequate remodeling by the active growth plates that are spared by the disease process [5, 34]. Some lesions may fail to respond or are unsuitable for treatment by injection because of their site, impending fracture, or soft-tissue invasion [33]. Furthermore, it seems that incomplete vertebral remodeling usually does not lead to chronic pain or compromise structural integrity [5, 24, 59, 60].

## Authors’ commentary and conclusion

This review summarizes current concepts in the diagnosis and management of patients with eosinophilic granuloma, with emphasis on the role of intralesional methylprednisolone injection for the successful cure of patients with symptomatic lesions. In the past, we planned for observation alone for patients with imaging evidence of eosinophilic granuloma, and curettage for the most painful lesions. It was our initial belief that percutaneous techniques do not provide adequate tissue for definitive diagnosis for mesenchymal tumors [61–63]. This belief was based on the agreement among pathologists that mesenchymal tumors are among the most difficult of pathologies to accurately diagnose. We then realized that patients, especially children with symptomatic bone lesions, should not be left alone for the disease to take its natural course without a histological diagnosis. Over the past 15 years, we have been able to refine the procedures for needle or trocar and frozen sections biopsy to assess the adequacy of the biopsy specimen. Nowadays, we believe that histological diagnosis is necessary for all bone lesions, and recommend that biopsy should not be considered as a strategy for treatment of eosinophilic granuloma but rather as a step to confirm diagnosis. By using CT-guided intralesional methylprednisolone injection, frozen sections histological diagnosis can be obtained in all patients. After biopsy, intralesional injection of methylprednisolone is considered beneficial [31–33], or at least not harmful. In our practice, tissue procurement and frozen sections biopsy are usually diagnostic in all patients with suspected eosinophilic granuloma. Even if the definite histological diagnosis is different, intralesional methylprednisolone injection would not have resulted in any adverse effect, but rather it would have decreased intralesional edema and provided pain relief. Our long-term results, (mean follow up, 9 years; range, 4–23 years) support biopsy and intralesional methylprednisolone injection as a safe treatment for eosinophilic granulomas of bone with complete resolution of pain and imaging reconstitution of the lesions.
